# Identification of lncRNAs associated with the pathogenesis of ankylosing spondylitis

**DOI:** 10.1186/s12891-021-04119-6

**Published:** 2021-03-12

**Authors:** Dan Huang, Jian Liu, Lei Wan, Yanyan Fang, Yan Long, Ying Zhang, Bingxi Bao

**Affiliations:** 1 Department of Rheumatology, First Affiliated Hospital of Anhui University of Chinese Medicine, No 117 Meishan Road, Shushan District, Hefei City, Anhui Province 230031 People’s Republic of China; 2 Rheumatology institute of Anhui Academy Chinese Medicine, No 117 Meishan Road, Shushan District, Hefei City, Anhui Province 230031 People’s Republic of China

**Keywords:** Ankylosing spondylitis, lncRNAs, mRNA, RNA-seq

## Abstract

**Background:**

Ankylosing spondylitis (AS) is a chronic autoimmune disease affecting the sacroiliac joint. To date, few studies have examined the association between long non-coding RNAs (lncRNAs) and AS pathogenesis. As such, we herein sought to characterize patterns of AS-related lncRNA expression and to evaluate the potential role played by these lncRNAs in this complex autoimmune context.

**Methods:**

We conducted a RNA-seq analysis of peripheral blood mononuclear cell (PBMC) samples isolated from five AS patients and corresponding controls. These data were then leveraged to characterize AS-related lncRNA expression patterns. We further conducted GO and KEGG enrichment analyses of the parental genes encoding these lncRNAs, and we confirmed the validity of our RNA-seq data by assessing the expression of six lncRNAs via qRT-PCR in 15 AS and control patient samples. Pearson correlation analyses were additionally employed to examine the associations between the expression levels of these six lncRNAs and patient clinical index values.

**Results:**

We detected 56,575 total lncRNAs in AS and control patient samples during our initial RNA-seq analysis, of which 200 and 70 were found to be up- and down-regulated (FC > 2 or < 0.05; *P* < 0.05), respectively, in AS samples relative to controls. In qRT-PCR validation assays, we confirmed the significant upregulation of NONHSAT118801.2, ENST00000444046, and NONHSAT183847.1 and the significant downregulation of NONHSAT205110.1, NONHSAT105444.2, and NONHSAT051856.2 in AS patient samples. We further found the expression of NONHSAT118801.2 and NONHSAT183847.1 to be positively correlated with disease severity.

**Conclusion:**

Overall, our findings highlight several lncRNAs that are specifically expressed in PBMCs of AS patients, indicating that they may play key functions in the pathogenesis of this autoimmune disease. Specifically, we determined that NONHSAT118801.2 and NONHSAT183847.1 may influence the occurrence and development of AS.

## Background

Ankylosing spondylitis (AS) is a chronic autoimmune inflammatory disease [1]. While AS is often asymptomatic during the early stages when it is most amenable to treatment, during the later stages of disease irreversible axial spine damage can develop, resulting in loss of mobility and debilitating pain in affected patients [2]. AS development is believed to be driven by numerous environmental and genetic factors [3], including macrophage activation status, infections with particular bacteria, and the expression of specific genes [4–6]. The exact etiology of this disease, however, remains incompletely characterized, and further in-depth analyses of the molecular basis of AS are thus warranted.

Long non-coding RNAs (lncRNAs) are RNAs > 200 nucleotides long that largely lack coding potential, and yet are critically important in physiological and pathological contexts [7]. Indeed, cell- and disease-specific lncRNA expression patterns have been characterized to date [8]. From a functional perspective, lncRNAs can control gene and protein expression at the epigenetic, transcriptional, and post-transcriptional levels via controlling genomic imprinting, chromatin remodeling, histone modification, splicing, transcription, and cell cycle progression [9, 10]. There is also evidence that lncRNAs are related to the incidence of autoimmune diseases including systemic lupus erythematosus [11–13], multiple sclerosis [14, 15], rheumatoid arthritis, and psoriasis [16–18]. The functional relevance of lncRNAs expressed in peripheral blood mononuclear cells (PBMCs) from AS patients, however, remain to be clarified.

Herein, we assessed AS-related lncRNA expression profiles via the RNA-seq analysis of PBMC samples from five AS patients and controls. We then used qRT-PCR to validate these RNA-seq findings. Together, our data offer a new theoretical framework for understanding the pathological basis of AS, highlighting novel disease-related lncRNAs for future research and therapeutic targeting efforts.

## Materials and methods

### Sample collection

We enrolled 15 total patients from the Department of Rheumatology at the First Affiliated Hospital of Anhui University of Chinese Medicine that had been diagnosed with AS based on the modified American College of Rheumatology (ACR) New York criteria (1984) [19]. We also enrolled 15 age- and sex-matched healthy controls. No enrolled patients had any history of prior diabetes, hepatitis, malignancies, cardiovascular disease, or other autoimmune/inflammatory diseases. All enrolled AS patients completed Bath ankylosing spondylitis diseases active index (BASDAI) [20], Bath ankylosing spondylitis functional index (BASFI) [21], and visual analog scale (VAS) assessments. In addition, we determined key clinical and laboratory parameters for each of these patients including erythrocyte sedimentation rate (ESR), C-reactive protein (CRP) levels, and immunoglobulin G (IgG) levels. We randomly selected five AS patients and five healthy controls for RNA sequencing analysis.

### PBMC preparation and total RNA extraction

Lymphoprep (Stemcell, USA) was utilized to separate PBMCs from the blood of AS patients and controls at room temperature via density gradient centrifugation. A MiRNeasy Mini Kit (Qiagen, Germany) was then used to extract total sample RNA, after which RNA quantity was evaluated using a NanoDrop 2000 instrument (Thermo Fisher Scientific, USA), and an Agilent 2100 bioanalyzer (Agilent Technologies, USA) was utilized to establish RNA quality [22].

### RNA-sequencing

A Truseq® chain total RNA sample preparation kit (Illumina, USA) was used to prepare RNA libraries based upon provided instructions, after which quantification was conducted using a Qubit 2.0 fluorometer, and quality was assessed via an Agilent 2100 Bioanalyzer. CBOT was then utilized to dilute the library to 10 pm in order to generate clusters, and an Illumina Hiseq 2500 instrument (Illumina) was utilized for sequencing. OG Biotech Inc. (AO Ji biotech, Shanghai, China) conducted all library preparation and sequencing.

### Data analysis

FastQC1 (v. 0.11.3) was initially utilized for quality control (QC) assessment of RNA-seq reads, after which rRNA reads, adapter sequences, and low-quality reads were trimmed with the seqtk2 software. BWA-MEM (v2.0.4) was then used to map the trimmed reads to the human reference genome (hg38), after which CIRI was used to predict lncRNAs in these RNA-seq data, and SRPBM was used to quantify lncRNA counts therein. Next, lncRNAs that were differentially expressed between AS and control patients were detected using the EdgeR software based on the following selection criteria: FC ≥ 2 or < 0.5; *P* < 0.05. The potential functional roles of these lncRNAs were assessed via GO and KEGG enrichment analyses of the parental genes harboring these differentially expressed lncRNAs. In addition, Pearson correlation analyses were used to detect associations between lncRNA and mRNA expression profiles, with a co-expression network being constructed by incorporating interactions with a Pearson’s R > 0.9 and *P* < 0.05. Cytoscape was then used to visualize this lncRNA-mRNA interaction network.

### qRT-PCR

To validate our RNA-seq data, we randomly selected three up-regulated (NONHSAT118801.2, ENST00000444046, and NONHSAT183847.1) and three down-regulated (NONHSAT205110.1, NONHSAT105444.2, and NONHSAT051856.2) lncRNAs from 270 differentially expressed lncRNAs via qRT-PCR in 15 AS and control samples with a Universal SYBR Green Master mix. OG-Biotech Inc. synthesized all primers used for this analysis (Table 1). The 2 − ΔΔCt method was used to assess relative lncRNA expression levels.

**Table 1:** Primers used in the present study

Gene	Primers sequences	pcr product length (bp)
GAPDH	F: ACAACTTTGGTATCGTGGAAGG	101
	R: GCCATCACGCCACAGTTTC	
NONHSAT118801.2	F: AGTCCCTGCTTTTAATTCTTTGGGG	155
	R: CAAGGAGGCAAACTCGGCTGC	
NONHSAT183847.1	F: GACACGGCTGCGTCCCTGAA	133
	R: GTGTGTGGTGGGCAGGGGAG	
ENST00000444046	F: CGACGGATCGGGAAAGCCAA	106
	R: GCCCGTTGTGAGCCTGAGAG	
NONHSAT205110.1	F: GGAGGCATGGGCTTGTCAAA	196
	R: CAAGCTTGTATTGCAGAAACTGT	
NONHSAT105444.2	F:CCACTCCGGGACATCTGCAC	81
	R: CCAGGCAGGTGGGTGTCAAC	
NONHSAT051856.2	F: GGGTCCTGCTTGTTGCCTGT	153
	R: GCTGTGGCTGTCCCAAACCT	

### Statistical analysis

GraphPad Prism 6.0 was used for the assessment of relative lncRNA expression and figure construction. Data are given as means ± standard deviation (SD), and were compared via Student’s t-tests. Relationships between different parameters were compared via Spearman and Pearson correlation analyses. *P* < 0.05 was the significance threshold for this study.

## Results

### Patient characteristics

We detected no significant differences in age or sex when comparing the AS and control patient cohorts in the present study (Table 2).

**Table 2:** Study population clinical characteristics

Indexes	AS	Control	*P* value
Sex (M/F)	11/4	11/4	1.00
Age (years)	33.60 ± 8.24	34.60 ± 7.03	0.723
Disease duration (years)	7.97 ± 6.18	NA	NA
ESR (mm/h)	51.27 ± 25.50	NA	NA
CRP (mg/dL)	55.30 ± 35.31	NA	NA
IgG (g/L)	13.34 ± 3.38	NA	NA
BASDAI (score)	6.55 ± 0.48	NA	NA
BASFI (score)	6.33 ± 0.56	NA	NA
VAS (score)	6.1 ± 1.09	NA	NA

### Identification of AS-related differentially expressed lncRNAs

**Fig. 1:**
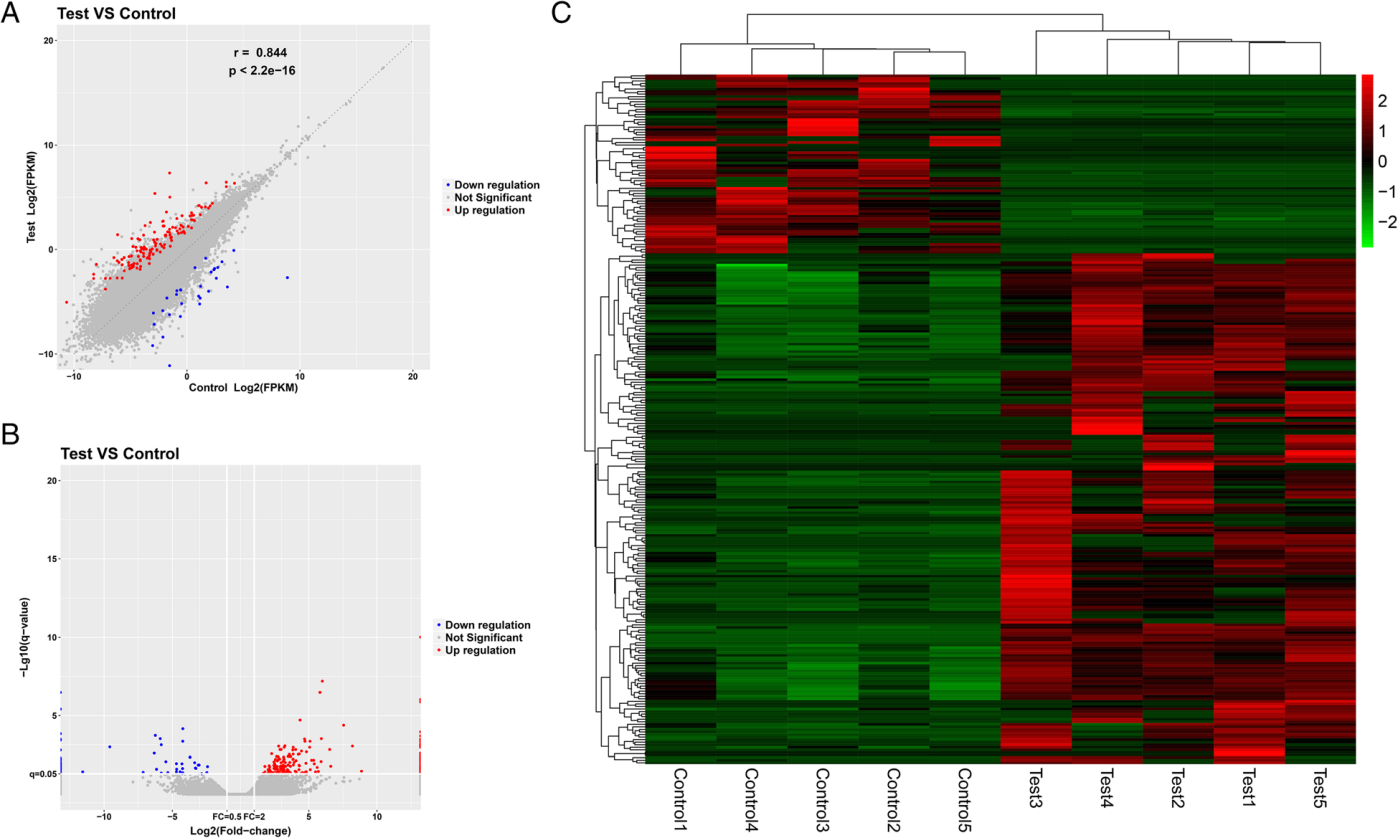
Scatter plot (**a**), Volcano plot (**b**), and Hierarchical clustering (**c**) analyses highlighting patterns of differential lncRNA expression in PBMC samples from AS patients and controls. Green and blue are used to indicate a > 2-fold reduction in the expression of the indicated lncRNAs in the AS group relative to controls in respective hierarchical clustering analyses and volcano plots (*P* < 0.05). Red corresponds to a > 2-fold increase in the expression of the indicated lncRNA in the AS group relative to the control group (*P* < 0.05)

We next identified lncRNAs that were differentially expressed between AS and control patient PBMCs using EdgeR (FC ≥ 2 or ≤ 0.5; *P* < 0.05). In total, we identified 270 lncRNAs that were differentially expressed between these two patient groups, of which 200 and 70 were up- and down-regulated in AS patient samples, respectively (Fig. 1a-c). The top 10 up- and down-regulated lncRNAs identified through these analyses are compiled in Tables 3 and 4.

**Table 3:** The top 10 known differentially expressed lncRNAs that were upregulated in AS patient PMBCs relative to control samples

lncRNAs	locus	length	log2FC	*P* value	Style
MSTRG.15374.10	14:49564494–49,586,878	1143	8.86469086799321	1.13E-04	Up
ENST00000592882	19:13153096–13,154,193	756	8.20263648791897	4.97E-07	Up
ENST00000520447	3:9388883–9,397,495	1809	7.55311232709341	6.47E-09	Up
NONHSAT242907.1	2:207176461–207,529,981	14,966	6.60740194542888	4.01E-05	Up
NONHSAT206983.1	6:26204366–26,204,807	442	6.53317269333968	1.08E-06	Up
MSTRG.38467.6	3:193667058–193,683,114	911	5.99499453964437	2.22E-12	Up
NONHSAT243832.1	20:40322988–40,323,673	686	5.9247373389848	8.16E-08	Up
NONHSAT226007.1	1:223992760–224,015,005	15,684	5.90517001896817	1.30E-05	Up
ENST00000574306	17:1711511–1,716,251	2626	5.8167471830444	2.33E-11	Up
NONHSAT233477.1	13:109765892–109,767,567	1529	5.70399695982728	1.21E-04	Up

**Table 4:** The top 10 known differentially expressed lncRNAs that were downregulated in AS patient PMBCs relative to control samples

lncRNAs	locus	length	log2FC	*P* value	Style
NONHSAT169063.1	14:22769653–22,799,551	5272	−2.42547013179437	4.36E-05	Down
NONHSAT213394.1	7:38239579–38,249,550	1035	−2.47555403789066	1.74E-04	Down
NONHSAT247151.1	3:195994009–195,997,627	3619	−2.8536961853987	2.31E-04	Down
NONHSAT123640.2	7:139430287–139,433,451	3165	−3.03849037973017	2.85E-05	Down
NONHSAT236815.1	16:81292454–81,314,778	22,325	−3.10565434683757	2.80E-05	Down
NONHSAT051856.2	16:717619–719,654	2036	−3.279800451942	6.36E-05	Down
NONHSAT248657.1	4:141296872–141,332,617	18,512	−3.31012780603255	5.80E-05	Down
NONHSAT229511.1	10:130061449–130,110,821	10,032	−3.37040958330436	1.70E-05	Down
NONHSAT230554.1	11:66472049–66,480,241	8193	−3.70572335168321	5.12E-06	Down
NONHSAT153273.1	1:244306789–244,309,614	2826	−4.23561043031746	1.21E-08	Down

### Pathway enrichment analyses of differentially expressed lncRNAs

In an effort to more fully explore the potential functional roles of these differentially expressed lncRNAs, we next conducted GO and KEGG enrichment analyses of the parental genes harboring these lncRNAs using the ClusterProfler tool.

**Fig. 2:**
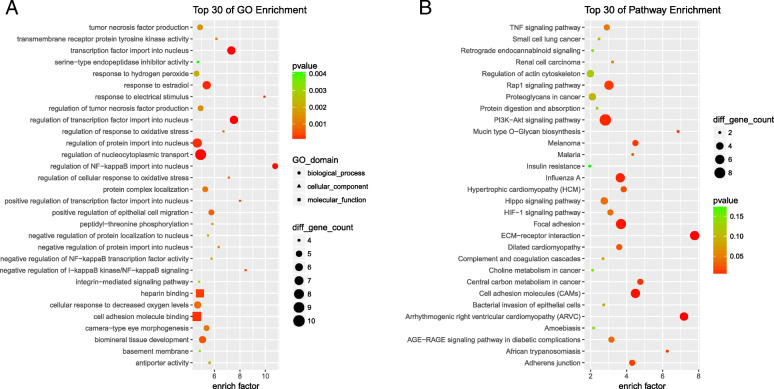
The results of GO and KEGG pathway enrichment analyses of parental genes harboring AS-related differentially expressed lncRNAs. **a** The top 30 enriched GO enrichment terms. **b** The top 30 classes of KEGG pathway enrichment terms

GO analyses enabled us to identify key biological processes (BPs), molecular functions (MFs), and cellular components (CCs) enriched among these lncRNAs (Fig. 2a). Significantly enriched BPs included the regulation of NF-κB nuclear import into the nucleus, responses to electrical stimuli, negative regulation of I-κB kinase/NF-κB signaling, and positive regulation of transcription factor import into the nucleus, while significantly enriched CC terms included basement membrane, and significantly enriched MFs included transmembrane receptor protein tyrosine kinase activity.

In a subsequent KEGG analysis, 134 pathways were found to be significantly enriched for these lncRNAs, with the top 30 being shown in Fig. 2b. The most significantly enriched of these pathways included the TNF, PI3K-Akt, HIF-1, Rap1, Hippo, ECM-receptor interaction, and arrhythmogenic right ventricular cardiomyopathy (ARVC) signaling pathways.

### Preparation of a lncRNA-mRNA co-expression network

**Fig. 3:**
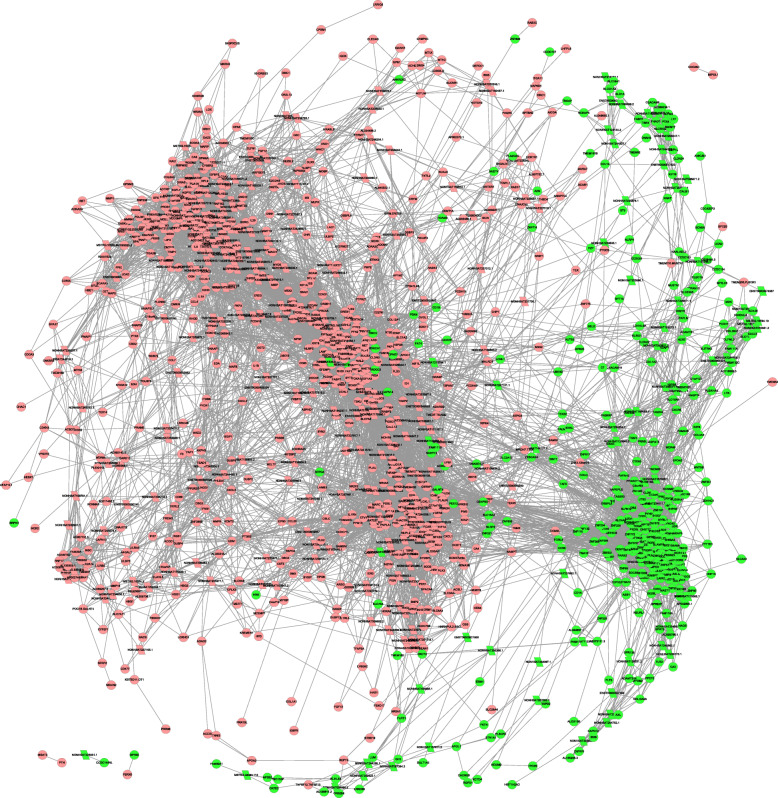
AS-related lncRNA-mRNA interaction network. An interaction network was constructed incorporating differentially expressed genes, with up- and down-regulated genes being marked in red and green, respectively

We next prepared a lncRNA-mRNA co-expression network using our RNA-seq data that incorporated pairs of lncRNAs and mRNAs that yielded Pearson correlation coefficient values ≥0.9, with Cytoscape being used to visualize this network. In total, 948 differentially expressed mRNA were obtained, including 628 upregulated expressed and 320 downregulated expressed. Co-expression networks generated using control and AS patient samples different substantially with respect to the number of incorporated nodes and connections (Fig. 3).

### RNA-seq result validation

**Fig. 4:**
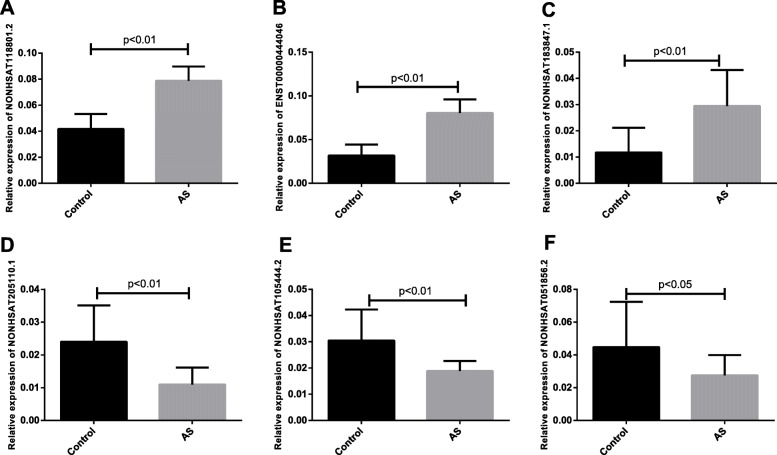
qRT-PCR-based validation of the expression of six differentially expressed lncRNAs in control and AS patient PBMCs

We next validated our RNA-seq data by assessing the expression of 6 randomly selected differentially expressed lncRNAs in PBMC samples from 15 AS and 15 control patients via qRT-PCR. This analysis enabled us to confirm that NONHSAT118801.2, ENST00000444046, and NONHSAT183847.1 were significantly upregulated in AS patient PMBCs, whereas NONHSAT205110.1, NONHSAT205110.1, and NONHSAT051856.2 were significantly downregulated in these samples (Fig. 4). The qRT-PCR results were consistent with the RNA-seq results.

### Correlations between lncRNA expression patterns and AS patient clinical findings

**Fig. 5:**
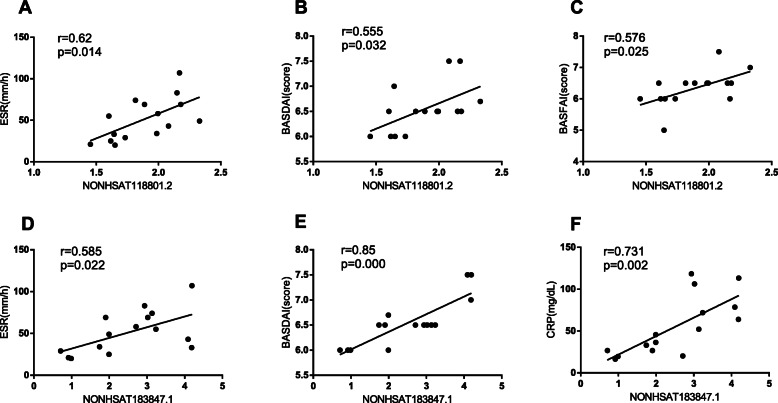
Correlations between lncRNA expression profiles and AS clinical disease activity

Lastly, we evaluated the association between the expression of the identified differentially expressed lncRNAs in AS patients and patient disease status. Specifically, we conducted Pearson correlation analyses comparing the expression of the six validated lncRNAs (NONHSAT118801.2, ENST00000444046, NONHSAT183847.1, NONHSAT205110.1, NONHSAT205110.1, and NONHSAT051856.2) and AS clinical features (ESR, hs-CRP, IgG, BASDAI, BASFAI, and VAS). We determined that NONHSAT118801.2 expression levels were positively correlated with ESR, BASDAI, and BASFAI levels in AS patients (Fig. 5a-c), while NONHSAT183847.1 levels were positively associated with ESR, BASDAI, and CRP (Fig. 5d-f).

## Discussion

While structurally similar to mRNAs, lncRNAs generally lack the ability to encode peptides. Although they were originally considered to be irrelevant and a form of transcriptional noise, lncRNAs are now recognized to be key regulators of gene and protein expression at the epigenetic, transcriptional, and post-transcriptional levels. These lncRNAs are thus key determinants of metabolic activity, development, evolution, and disease pathophysiology [22].

A previous microarray study identified five lncRNAs, NDRG1-AS6, CSNK1D-AS8, CD46-AS9, SMYD5-AS2, and NR_045553 that are involved in hip joint ligament tissues in patients with AS [23]. In addition, MSTRG.8559 and LINC00987 were identified as two hub differentially expressed lncRNAs in AS patients [24]. In one recent study, peripheral blood lncRNA-AK001085 levels were found to be reduced in AS patients and to be negatively correlated with CRP and ESR levels in these individuals, suggesting a potential protective role for this lncRNA in this pathological context [25]. Although the role of LncRNAs in the pathogenesis of AS has been preliminarily understood, the regulatory mechanism of most LncRNA in AS remains unclear due to its large system, complex mechanism and diverse functions. Our data provide novel insights into lncRNA expression profiles in AS patient PBMCs, as we were able to identify 270 total differentially expressed lncRNAs, including 200 and 70 that were up- and down-regulated in AS patient samples, respectively. We validated the differential expression of three up- and down-regulated lncRNAs via qRT-PCR to confirm the reliability of our RNA-seq data, The qRT-PCR results were consistent with the RNA-seq resultsand selected these lncRNAs for further analysis. Together, our data have the potential to serve as a framework for future studies of the role of these lncRNAs in the context of AS disease processes.

Our GO analysis results indicate potential roles for these differentially expressed lncRNAs as important regulators of NF-κB, which is a key transcription factor and regulator of inflammatory and oxidative stress-related processes in cells. These lncRNAs were also associated with signaling pathways including the TNF, PI3K-Akt, Rap1, Hippo, and HIF-1 pathways, which are primarily involved in inflammatory immune regulation and osteogenic differentiation. In some prior studies, the PI3K-Akt signaling pathway has been suggested to promote fibroblastic ossification and inflammation in the context of AS [26]. Some of the identified pathways, including the Rap1, Hippo, and HIF-1 pathways, have not previously been studied in the context of AS, and thus warrant future investigation.

To confirm our RNA-seq findings, we utilized a qRT-PCR approach to validate the differential expression of three up-regulated lncRNAs (NONHSAT118801.2, ENST00000444046, NONHSAT183847.1) and three down-regulated lncRNAs (NONHSAT205110.1, NONHSAT205110.1, NONHSAT051856.2) in 15 AS patient samples relative to 15 control samples. This approach confirmed that all six of these lncRNAs were differentially expressed in the expected directions (all *P* < 0.01), thereby reaffirming the reliability of our RNA-seq data. We also assessed correlations between the expression levels of these lncRNAs and AS patient disease indices, leading us to determine that NONHSAT118801.2 expression was positively correlated with ESR, BASDAI, and BASFAI levels in AS (Fig. 5a-c), while NONHSAT183847.1 expression was positively correlated with ESR, BASDAI, and CRP. These data suggest that NONHSAT118801.2 and NONHSAT183847.1 may be key regulators of the pathogenesis of AS, although additional research will be necessary in order to understand the mechanisms whereby these candidate lncRNAs impact disease progression.

Together, our data offer a new mechanistic basis for understanding the pathogenesis of AS. However, we recognized that the present study has some limitations. First, the sample size was small, necessitating the collection of a larger sample from different regions and involving subjects belonging to different races. Second, we did not assess the ability of the markers to effectively differentiate AS from other rheumatic diseases such as Rheumatoid Arthritis (RA), systemic lupus erythematosus (SLE), and multiple sclerosis (MS). These other rheumatic diseases should be considered in similar future studies so as to strengthen the argument supporting that differentially expressed lncRNAs are useful diagnostic biomarkers for AS.

## Conclusion

In summary, we utilized an RNA-seq approach to characterize AS-related lncRNA expression profiles in patient PBMCs. The identified differentially expressed lncRNAs may offer new insight into the molecular etiology of this complex and debilitating disease. However, future mechanistic analyses will be required in order to validate our findings and to fully explore the clinical and therapeutic relevance of these lncRNAs in this disease.

## Data Availability

The datasets generated and/or analysed during the current study are available in National Genomics Data Center (Nucleic Acids Res 2021), China National Center for Bioinformation/Beijing Institute of Genomics, Chinese Academy of Sciences, under accession number PRCA004516 that are publicly accessible at https://bigd.big.ac.cn/gsa.

## Abbreviations

AS: Ankylosing spondylitis
lncRNAs: Long non-coding RNAs;
RNA-seq: High-throughput RNA sequencing
PBMC: Peripheral blood mononuclear cell
qRT-PCR: Quantitative real-time polymerase chain reaction

## References

[1] Liu G, Ma Y, Yang Q, Deng S (2020). Modulation of inflammatory response and gut microbiota in ankylosing spondylitis mouse model by bioactive peptide IQW. J Appl Microbiol.

[2] Ghasemi-Rad M, Attaya H, Lesha E, Vegh A, Maleki-Miandoab T, Nosair E, Sepehrvand N, Davarian A, Rajebi H, Pakniat A (2015). Ankylosing spondylitis: a state of the art factual backbone. World J Radiol.

[3] Ermann J (2020). Pathogenesis of axial Spondyloarthritis — sources and current state of knowledge. Rheum Dis Clin N Am.

[4] Sveaas SH, Berg IJ, Provan SA, Semb AG, Olsen IC, Ueland T, Aukrust P, Vøllestad N, Hagen KB, Kvien TK (2015). Circulating levels of inflammatory cytokines and cytokine receptors in patients with ankylosing spondylitis: a cross-sectional comparative study. Scand J Rheumatol.

[5] Rashid T, Ebringer A (2006). Ankylosing spondylitis is linked to Klebsiella—the evidence. Clin Rheumatol.

[6] Zeng L, Lindstrom MJ, Smith JA (2011). Ankylosing spondylitis macrophage production of higher levels of interleukin-23 in response to lipopolysaccharide without induction of a significant unfolded protein response. Arthritis Rheum.

[7] Mercer TR, Dinger ME, Mattick JS (2009). Long non-coding RNAs: insights into functions. Nat Rev Genet.

[8] Aune TM, Crooke PS, Patrick AE, Tossberg JT, Olsen NJ, Spurlock CF (2017). Expression of long non-coding RNAs in autoimmunity and linkage to enhancer function and autoimmune disease risk genetic variants. J Autoimmun.

[9] Li J, Li Z, Leng K, Xu Y, Ji D, Huang L, Cui Y, Jiang X (2018). ZEB1-AS1: A crucial cancer-related long non-coding RNA. Cell Prolif.

[10] Huang Z, Zhou J-K, Peng Y, He W, Huang C (2020). The role of long noncoding RNAs in hepatocellular carcinoma. Mol Cancer.

[11] Xu H, Chen W, Zheng F, Tang D, Liu D, Wang G, Xu Y, Yin L, Zhang X, Dai Y (2020). Reconstruction and analysis of the aberrant lncRNA–miRNA–mRNA network in systemic lupus erythematosus. Lupus..

[12] Cao HY, Li D, Wang YP, Lu HX, Sun J, Li HB (2020). Clinical significance of reduced expression of lncRNA TUG1 in the peripheral blood of systemic lupus erythematosus patients. Int J Rheum Dis.

[13] Li J, Wu G-C, Zhang T-P, Yang X-K, Chen S-S, Li L-J, Xu S-Z, Lv T-T, Leng R-X, Pan H-F (2017). Association of long noncoding RNAs expression levels and their gene polymorphisms with systemic lupus erythematosus. Sci Rep.

[14] Ghaiad HR, Elmazny AN, Nooh MM, El-Sawalhi MM, Shaheen AA (2019). Long noncoding RNAs APOA1-AS, IFNG-AS1, RMRP and their related biomolecules in Egyptian patients with relapsing-remitting multiple sclerosis: relation to disease activity and patient disability. J Adv Res.

[15] Moradi A, Naiini MR, Yazdanpanahi N, Tabatabaeian H, Nabatchian F, Baghi M, Azadeh M, Ghaedi K (2020). Evaluation of The Expression Levels of Three Long Non-Coding RNAs in Multiple Sclerosis. Cell J (Yakhteh).

[16] Yan S, Wang P, Wang J, Yang J, Lu H, Jin C, Cheng M, Xu D (2019). Long non-coding RNA HIX003209 promotes inflammation by sponging miR-6089 via TLR4/NF-κB signaling pathway in rheumatoid arthritis. Front Immunol.

[17] Yuan M, Wang S, Yu L, Qu B, Xu L, Liu L, Sun H, Li C, Shi Y, Liu H (2017). Long noncoding RNA profiling revealed differentially expressed lncRNAs associated with disease activity in PBMCs from patients with rheumatoid arthritis. PLoS One.

[18] Zhou Q, Yu Q, Gong Y, Liu Z, Xu H, Wang Y, Shi Y (2019). Construction of a lncRNA-miRNA-mRNA network to determine the regulatory roles of lncRNAs in psoriasis. Exp Ther Med.

[19] Linden SVD, Valkenburg HA, Cats A (1984). Evaluation of diagnostic criteria for ankylosing spondylitis. Arthritis Rheum.

[20] Garrett S, Jenkinson T, Kennedy LG, Whitelock H, Gaisford P, Calin A (1994). A new approach to defining disease status in ankylosing spondylitis: the Bath ankylosing spondylitis disease activity index. J Rheumatol.

[21] Calin A, Garrett S, Whitelock H, Kennedy LG, O’Hea J, Mallorie P, Jenkinson T (1994). A new approach to defining functional ability in ankylosing spondylitis: the development of the Bath ankylosing spondylitis functional index. J Rheumatol.

[22] Ruiz-Orera J, Messeguer X, Subirana JA, Alba MM (2014). Long non-coding RNAs as a source of new peptides. Elife..

[23] Xu Z, Zhou X, Hao L, Chen Q, Chen G (2019). Identification of the key genes and long non-coding RNAs in ankylosing spondylitis using RNA sequencing. Int J Mol Med.

[24] Zhang C, Wang C, Jia Z, Tong W, Liu D, He C, Huang X, Xu W (2017). Differentially expressed mRNAs, lncRNAs, and miRNAs with associated co-expression and ceRNA networks in ankylosing spondylitis. Oncotarget.

[25] Li X, Chai W, Zhang G, Ni M, Chen J, Dong J, Zhou Y, Hao L, Bai Y, Wang Y (2017). Down-regulation of lncRNA-AK001085 and its influences on the diagnosis of ankylosing spondylitis. Med Sci Monit.

[26] Qin X, Jiang T, Liu S, Tan J, Wu H, Zheng L, Zhao J (2017). Effect of metformin on ossification and inflammation of fibroblasts in ankylosing spondylitis: an in vitro study. J Cell Biochem.

